# Intestinal Stem Cell Marker ASCL2 is a Novel Prognostic Predictor in Esophageal Adenocarcinoma

**DOI:** 10.7759/cureus.21021

**Published:** 2022-01-07

**Authors:** Yukiko Shibahara, Osvaldo Espin-Garcia, James Conner, Jessica Weiss, Mathieu Derouet, Jonathan Allen, Frances Allison, Sangeetha Kalimuthu, Jonathan C Yeung, Gail E Darling

**Affiliations:** 1 Latner Thoracic Surgery Research Laboratories, University Health Network, Toronto, CAN; 2 Department of Pathology, Laboratory Medicine Program, University Health Network, University of Toronto, Toronto, CAN; 3 Department of Pathology, Kitasato University, School of Medicine, Sagamihara, JPN; 4 Department of Biostatistics, Princess Margaret Cancer Centre, University Health Network, Toronto, CAN; 5 Division of Biostatistics, Dalla Lana School of Public Health, University of Toronto, Toronto, CAN; 6 Department of Pathology and Laboratory Medicine, Mount Sinai Hospital, University of Toronto, Toronto, CAN; 7 Division of Thoracic Surgery, Department of Surgery, Toronto General Hospital, Toronto, CAN; 8 Division of Thoracic Surgery, Department of Surgery, University Health Network, University of Toronto, Toronto, CAN

**Keywords:** wnt pathway, lgr5, ascl2, intestinal stem cell, barrett's esophagus, esophageal adenocarcinoma

## Abstract

Purpose

Intestinal stem cell markers play a significant role in esophageal adenocarcinoma carcinogenesis via Barrett’s esophagus; however, its utility as a prognostic biomarker has not been established.

Methods

We analyzed the immunohistochemical expression of intestinal stem cell markers, ASCL2 and LGR5, using whole slides (35 cases) and tissue microarray (TMA; 64 cases). On TMA slides, adjacent normal squamous epithelium, metaplastic glandular epithelium (Barrett's esophagus), and dysplastic glandular epithelium were inserted when applicable. Two pathologists semi-quantitatively scored stained slides independently, and the results were correlated with clinicopathologic factors and outcomes.

Results

In whole slides, 51% and 57% expressed high ASCL2 and high LGR5; in TMA, 69% and 88% expressed high ASCL2 and high LGR5, respectively. In TMA, high ASCL2 and low LGR5 expression significantly correlated to a higher number of involved lymph nodes (p=0.027 and p=0.0039), and LGR5 expression significantly correlated to the pathological stage (p=0.0032). Kaplan-Meier analysis showed a negative impact of high ASCL2 expression on overall survival (OS; WS p=0.0168, TMA p=0.0276) as well as progression-free survival (PFS; WS p=0.000638, TMA p=0.0466) but not LGR5. Multivariate Cox regression analysis revealed that ASCL2 expression is an independent prognostic factor for esophageal adenocarcinoma (OS; WS p=0.25, TMA p=0.011. PFS; WS p=0.012, TMA p=0.038). Analysis of the TCGA dataset showed that ASCL2 mRNA levels were correlated to nodal status but not overall survival.

Conclusion

High expression of the intestinal stem cell marker ASCL2 may predict unfavorable outcomes in surgically resected esophageal adenocarcinoma.

## Introduction

Esophageal adenocarcinoma (EAC) is one of the least studied cancers and is currently the sixth-leading cause of cancer death globally [1]. The epidemiology of EAC is striking in that it is the dominant histological type in the Western world, and its incidence is steadily continuing to increase [2]. The major pathway to EAC involves gastroesophageal reflux disease resulting in Barrett's esophagus (BE) and subsequent EAC in 0.5%-1%. Glandular dysplasia is commonly observed during this process between BE and EAC [1]. BE is characterized by the replacement of normal squamous epithelium of the lower portion of the esophagus with intestinal metaplasia. While BE is the primary precursor lesion and a potent risk factor of EAC, the developmental process from BE to EAC takes about three decades and why some BE progress to EAC but others do not is unknown [3-4].

There has been extensive research in colorectal carcinoma, surrounding the roles of intestinal stem cells (ISCs) in its development and metastasis [5-6]. ISCs found at the base of intestinal crypts express Leucine-rich-repeat-containing G-protein-coupled receptor 5 (LGR5), a molecular marker that sustains the self-renewing function of ISCs [7]. Recent studies demonstrated LGR5 positive cells to make up a subpopulation of cancer stem cells (CSCs), proving the dual role of LGR5 to regulate "stemness" in both healthy ISCs and CSCs [8]. LGR5 upregulation is observed in various cancers, including ISC-associated cancers, such as colorectal and stomach carcinoma [9-10], and this upregulation leads to the activation of the Canonical Wnt signaling pathway [11]. Altered Wnt signaling plays a dominant role in BE progression [12], and its activation is shown to promote dysplastic change [13].

Gene profiling of LGR5 positive ISCs led to the discovery of Achaete scute-like 2 (ASCL2), which is a basic helix-loop-helix transcription factor. Transgenic manipulation of ASCL2 revealed that ISC fate relies on this expression [14]. ASCL2 is also a downstream target of Wnt signaling [15], which is critical in the carcinogenesis and progression of EAC.

Considering that BE not only resembles the intestinal mucosa morphologically but also possesses an ISC population within [16-17], we hypothesized that key ISC markers, LGR5 and ASCL2, have clinicopathological significance in EAC. We approached this using immunohistochemistry (IHC) of whole slides (WS) and tissue microarray (TMA) and validated it with TCGA expression data.

This article was previously presented as a meeting abstract (e-poster) at the 17th World Congress for Esophageal Diseases (ISDE 2021) online (September 27-30, 2021). This article was previously posted to the ResearchGate preprint server on March 10, 2021.

## Materials and methods

Patient selection

Patients diagnosed with EAC at University Health Network, Toronto, ON, Canada, between 2001 and 2011 were included in our study. Locally advanced as well as distant metastatic patients were enrolled and all cases were surgically resected. All methods were performed in accordance with relevant guidelines/regulations. University Health Network Research Ethics Board (CAPCR/UHN REB number 13-6551) approved, and all participants provided written informed consent to have their surgical specimens banked and used in future research. Clinicopathological information (age, gender, pathological stage, histological grade, neoadjuvant therapy, and positive lymph nodes) were obtained from electronic hospital records, and the original hematoxylin and eosin (H&E) stained slides were reviewed by two pathologists. Histological variants of adenocarcinoma and other types of carcinoma were excluded from the study.

WS preparation and TMA construction

Thirty-five cases were available for WS IHC. WS sections were cut at the 4-μm interval, and unstained slides were prepared for IHC.

Sixty-four cases were available for TMA block construction. After verification with H&E staining, representative tumor areas of up to three areas, 0.6 mm in diameter, were selected and deposited into a paraffin block with a tissue-array instrument (Beecher Instruments, Silver Springs, MD). When applicable, the normal squamous epithelium was inserted adjacent to tumor samples as negative control; BE (metaplastic glandular epithelium) and dysplastic glandular epithelium were inserted for comparison. Consecutive 4-μm unstained TMA sections were cut and placed on slides for IHC analysis.

Immunohistochemistry

Paraffin-embedded sections were deparaffinized in xylene and rehydrated in an alcohol series. Blocking was performed using hydrogen peroxide (3%, 10 minutes). Antigen retrieval was performed in the Decloaker solution (pH9) using a Decloaking Chamber (Biocare Medical, Pacheco, CA). The slides were incubated overnight at 4 degrees Celsius, with primary antibodies anti-ASCL2 (R&D systems, cat#AF6539, 1:200) and anti-LGR5 (Abcam, cat#75850, 1:200). After washing with phosphate-buffered saline with Tween (PBST), the slides were incubated with a secondary antibody for 60 minutes and then washed again with PBST. Diaminobenzidine (DAB) was used as a chromogen, and nuclear counterstaining was performed with hematoxylin. Then slides were dehydrated through graded alcohols, cleared in xylene, and coverslipped. For positive control, normal colon tissue was used; for negative control, we used phosphate-buffered saline (PBS) instead of the primary antibody.

Immunohistochemical analysis

IHC scores were evaluated by two pathologists independently, and both pathologists were blinded from clinical data. The scoring system was based on previously published literature [18-19]. Briefly, the expected staining pattern for ASCL2 was cytoplasmic and LGR5 was cytoplasmic or membranous [18-19]. Each was semiquantitatively scored for staining intensity (1 - weak; 2 - moderate; 3 - strong) and the percentage of positive staining cells (0, <10%; 1, 11-30%; 2, 31-50%; 3, 51-80%; 4, >80%) [18-19]. All final IHC scores were calculated by multiplying the two factors with a cut-off value of 4 [18-19]. If scores for the two samples were discordant, the final score for the tumor was upgraded to the higher score.

Follow-up

For survival analysis, disease-free survival (DFS) and overall survival (OS) were calculated. Two patients who died within six months of surgery due to post-surgical complications were excluded from both WS and TMA overall survival analysis.

Statistical analysis 

All data were analyzed with R version 3.5.2 (The R Foundation for Statistical Computing, Vienna, Austria). Univariable and multivariable statistical analyses were performed to determine the association among ASCL2/LGR5 expression levels, clinicopathological features, and time-to-event outcomes (OS and PFS). Differences in ASCL2/LGR5 expression levels were assessed in univariable analysis using Fisher's exact tests on categorical variables and Wilcoxon-Mann-Whitney tests on continuous variables. Age, gender, histological grade, number/positivity of metastatic lymph nodes, pathological stage, and neoadjuvant therapy were considered potential confounding factors. The Kaplan-Meier (KM) method and the Cox proportional hazards regression (Cox PH) were performed to investigate the association between ASCL2/LGR5 expression levels and time-to-event outcomes. For KM, differences in survival curves were ascertained by the log‐rank test. In Cox PH, both adjusted and unadjusted models were fitted. Gender was excluded from the WS Cox PH analysis, as there were no such survival events among female patients in the WS cohort. A p-value of less than 0.05 was considered statistically significant.

Bioinformatic analysis

To validate the prognostic value of ASCL2 in EAC, mRNA and clinical profile data were downloaded from the online cBioPortal for Cancer Genomics (http://www.cbioportal.org/. date last accessed, January 29, 2020) [20-21]. Data from PanCancer Atlas was used [22]. The cBioportal was also used to analyze the alteration frequency of ASCL2 mutations in EAC.

## Results

ASCL2 and LGR5 expression on whole slides

The clinicopathological characteristics of 35 cases on WS are summarized in Table 1. Briefly, the age range was 38 to 78 years old (mean age of 65.5 years), and males were the predominant sex (89%). Twenty percent (20%) of the patients received neoadjuvant chemotherapy. The pathological stage was based on the 8th edition AJCC/UICC staging of cancers of the esophagus and esophagogastric junction [23].

**Table 1:** Clinicopathological characteristics and ASCL2 and LGR5 expression (whole slides) ASCL2, Achaete scute-like 2; LGR5, Leucine-rich-repeat-containing G-protein-coupled receptor 5; ASCL2^low^, cases with low ASCL2 expression; ASCL2^high^, cases with high ASCL2 expression; LGR5^low^, cases with low LGR5 expression; LGR5^high^, cases with high LGR5 expression; sd, standard deviation; Min, minimum; Max, maximum

Covariate		Full sample (n=35)	ASCL2^low^ (n=17)	ASCL2^high^ (n=18)	p-value	LGR5^low^ (n=20)	LGR5^high^ (n=15)	p-value
Age	Mean (sd)	65.5 (10.6)	66.5 (10)	64.5 (11.3)	0.69	65.5 (10.8)	65.5 (10.6)	0.93
	Median (range)	66 (38-78)	66 (38-78)	65.5 (41-78)		68 (38-78)	64 (41-78)	
Gender	Female	4 (11)	3 (18)	1 (6)	0.34	2 (10)	2 (13)	1
	Male	31 (89)	14 (82)	17 (94)		18 (90)	13 (87)	
Stage	I/II	6 (17)	4 (24)	2 (11)	0.18	3 (15)	3 (20)	1
	III	20 (57)	11 (65)	9 (50)		12 (60)	8 (53)	
	IV	9 (26)	2 (12)	7 (39)		5 (25)	4 (27)	
Neoadjuvant chemotherapy	No	28 (80)	15 (88)	13 (72)	0.4	15 (75)	13 (87)	0.67
	Yes	7 (20)	2 (12)	5 (28)		5 (25)	2 (13)	
Number of positive nodes	Mean (sd)	5.6 (8.3)	3.4 (3.2)	7.7 (10.9)	0.22	4.7 (5.4)	6.8 (11.2)	0.89
	Median (Min,Max)	4 (0,45)	3 (0,11)	4 (0,45)		4 (0,24)	4 (0,45)	
Number of sampled nodes	Mean (sd)	23 (11.9)	25.7 (12.8)	20.4 (10.7)	0.35	20.6 (10.1)	26.1 (13.6)	0.2
	Median (Min,Max)	20 (0,49)	21 (9,49)	19.5 (0,45)		18.5 (6,49)	21 (0,46)	
Histological grade	G1	1 (3)	1 (6)	0 (0)	0.086	0 (0)	1 (7)	0.48
	G2	20 (57)	12 (71)	8 (44)		11 (55)	9 (60)	
	G3	14 (40)	4 (24)	10 (56)		9 (45)	5 (33)	

**Figure 1:**
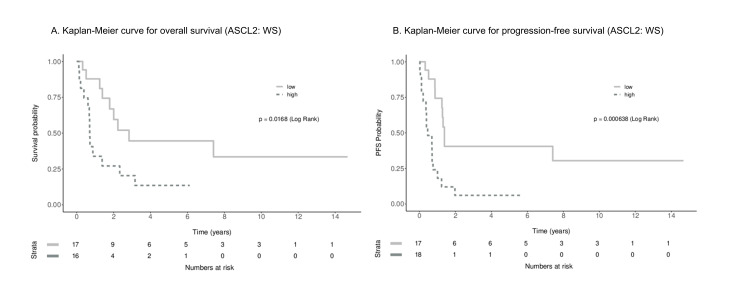
Survival analysis of 35 esophageal adenocarcinoma whole slides by Kaplan-Meier curve A. The overall survival (OS) rate in patients with high ASCL2 expression (dashed line) was significantly lower than that in patients with low ASCL2 expression (solid line), B. Progression-free survival (PFS) in patients with high ASCL2 expression (dashed line) was significantly lower than that in patients with low ASCL2 expression (solid line) ASCL2, Achaete scute-like 2; WS, whole slides; PFS, progression-free survival.

Based on IHC scores, 18 cases (51%) were ASCL2^high^ and 17 cases (49%) were ASCL2^low^. ASCL2 was localized to the cytoplasm of tumor cells. ASCL2 expression tended to be correlated to histological grade (p=0.086) but did not have any significant correlation with any other clinicopathological factors. The percentage of ASCL2^high^ is 33.3% in pathological Stage I/II, 45% in Stage III, and 77.8% in Stage IV but did not reach statistical significance due to the small number of Stage I/II cases in our study. For our study population (WS), the median overall survival (OS) time was 1.8 years (95% CI,0.7-7.4) and progression-free survival (PFS) time was 0.9 years (95% CI, 0.7-1.4). ASCL2 showed statistical significance in the KM analyses for OS and PFS, p=0.0168, and p=0.000638, respectively (Figure 1).

Univariable Cox PH analyses showed ASCL2 significance in both OS and PFS (OS HR=2.83, 95% CI=1.16-6.9, p=0.022; PFS HR=3.93, 95% CI=1.69-9.12, p=0.0014). In multivariate analysis adjusting for pathological stage, ASCL2 remained statistically significant for PFS (HR=3.13, 95% CI=1.28-7.65, p=0.012) but not for OS (HR=1.78, 95% CI=0.66-4.76, p=0.25) (Tables 2-3).

**Table 2:** Univariable and multivariable analysis of overall survival (whole slides) p<0.05 was considered statistically significant and is highlighted in *italics*. ASCL2, Achaete scute-like 2; LGR5, Leucine-rich-repeat-containing G-protein-coupled receptor 5; HR, hazard ratio; CI, confidence interval

Covariate		HR (95% CI)	p-value	Global p-value
Univariable	n=33	22 events		
Age		1.01 (0.97,1.05)		0.69
Histological grade	G1	Reference		0.31
	G2	0.61 (0.08,4.85)	0.64	
	G3	1.23 (0.15,9.89)	0.85	
Stage	I/II	Reference		<0.001
	III	2.22 (0.49,9.93)	0.041	
	IV	23.95 (3.93,146.05)	<0.001	
Node positive		1.07 (1.03,1.11)		0.001
ASCL2 score	Low	Reference		0.022
	High	2.83 (1.16,6.9)		
LGR5 score	Low	Reference		0.5
	High	0.74 (0.31,1.77)		
Multivariable	n=33	22 events		
ASCL2 score	Low	Reference		0.25
	High	1.78 (0.66,4.76)		
Stage	I/II	Reference		0.0025
	III	2.18 (0.49,9.78)	0.31	
	IV	17.92 (2.81,114.28)	0.0023	

**Table 3:** Univariable and multivariable analysis of progression-free survival (whole slides) p<0.05 was considered statistically significant and is highlighted in *italics*. ASCL2, Achaete scute-like 2; LGR5, Leucine-rich-repeat-containing G-protein-coupled receptor 5; HR, hazard ratio; CI, confidence interval

Covariate	Covariate	HR (95%CI)	p-value	Global p-value
Univariable	n=35	26 events		
Age		0.99 (0.96,1.03)		0.73
Stage	I/II	Reference		0.0045
	III	1.47 (0.42,5.08)	0.55	
	IV	9.15 (1.98,42.27)	0.0046	
Node positive		1.05 (1.01,1.09)		0.0065
ASCL2 score	Low	Reference		0.0014
	High	3.93 (1.69,9.12)		
LGR5 score	Low	Reference		0.48
	High	0.75 (0.34,1.66)		
Multivariable	n=35	26 events		
ASCL2 score	low	Reference		0.012
	high	3.13 (1.28,7.65)		
Stage	I/II	Reference		0.035
	III	1.21 (0.34,4.3)	0.77	
	IV	5.4 (1.15,25.33)	0.033	

LGR5 was localized to the cytoplasm and membrane of tumor cells. Twenty cases (57%) were LGR5^high^ and 15 cases (43%) were LGR5^low^. LGR5 was not significantly correlated to any of the clinicopathological factors. LGR5 did not show statistical significance in KM analyses for survival (OS: p=0.449, PFS: p=0.459; Data not shown). Univariable Cox PH analyses also failed to show LGR5 significance in OS and PFS (Tables 2-3: OS HR=0.74, 95% CI=0.31-1.77, p=0.5; PFS HR=0.75, 95% CI=0.34-1.66, p=0.48).

ASCL2 and LGR5 expression on TMA slides

Sixty-four EAC patients were included in the TMA slides. Briefly, the age range was 38 to 86 years old (mean age of 64.6 years), and males were the predominant sex (84%). Thirty-one percent (31%) of the patients received neoadjuvant chemotherapy (Table 4).

**Table 4:** Clinicopathological characteristics and ASCL2 and LGR5 expression (tissue microarray) p<0.05 was considered statistically significant and is highlighted in *italics*. ASCL2, Achaete scute-like 2; LGR5, Leucine-rich-repeat-containing G-protein-coupled receptor 5; ASCL^low^, cases with low ASCL2 expression; ASCL2^high^, cases with high ASCL2 expression; LGR5^low^, cases with low LGR5 expression; LGR5^high^, cases with high LGR5 expression; sd, standard deviation; Min, minimum; Max, maximum.sd, standard deviation; Min, minimum; Max, maximum

Covariate		Full sample (n=64)	ASCL2^low^ (n=20)	ASCL2^high^ (n=44)	p-value	LGR5^low ^(n=8)	LGR5^high^ (n=56)	p-value
Age	Mean (sd)	64.6 (11.4)	64.1 (12.7)	64.8 (10.8)	0.83	65.9 (9.6)	64.4 (11.7)	0.84
	Median (range)	64.5 (38-86)	64 (44-86)	65.5 (38-84)		69 (52-76)	64 (38-86)	
Gender	Female	10 (16)	6 (30)	4 (9)	0.059	1 (12)	9 (16)	1
	Male	54 (84)	14 (70)	40 (91)		7 (88)	47 (84)	
Stage	I/II	13 (20)	6 (30)	7 (16)	0.48	1 (12)	12 (21)	0.0032
	III	34 (53)	9 (45)	25 (57)		1 (12)	33 (59)	
	IV	17 (27)	5 (25)	12 (27)		6 (75)	11 (20)	
Neoadjuvant chemotherapy	No	44 (69)	12 (63)	32 (71)	0.56	7 (88)	37 (66)	0.42
	Yes	20 (31)	7 (37)	13 (29)		1 (12)	19 (34)	
Nodes positive	Mean (sd)	5.1 (7.2)	3.5 (5.4)	5.8 (7.8)	0.027	12.2 (14.5)	4.1 (4.9)	0.039
	Median (Min,Max)	3 (0,45)	1 (0,18)	4 (0,45)		8 (0,45)	3 (0,24)	
Nodes sampled	Mean (sd)	22.5 (10.9)	21.6 (11.5)	23 (10.7)	0.94	24.4 (13.7)	22.3 (10.6)	0.66
	Median (Min,Max)	21.5 (0,49)	22 (0,40)	21.5 (2,49)		26.5 (6,45)	21 (0,49)	
Histological grade	G1	3 (5)	1 (5)	2 (5)	0.27	0 (0)	3 (5)	0.82
	G2	37 (58)	9 (45)	28 (64)		4 (50)	33 (59)	
	G3	24 (38)	10 (50)	14 (32)		4 (50)	20 (36)	

**Figure 2:**
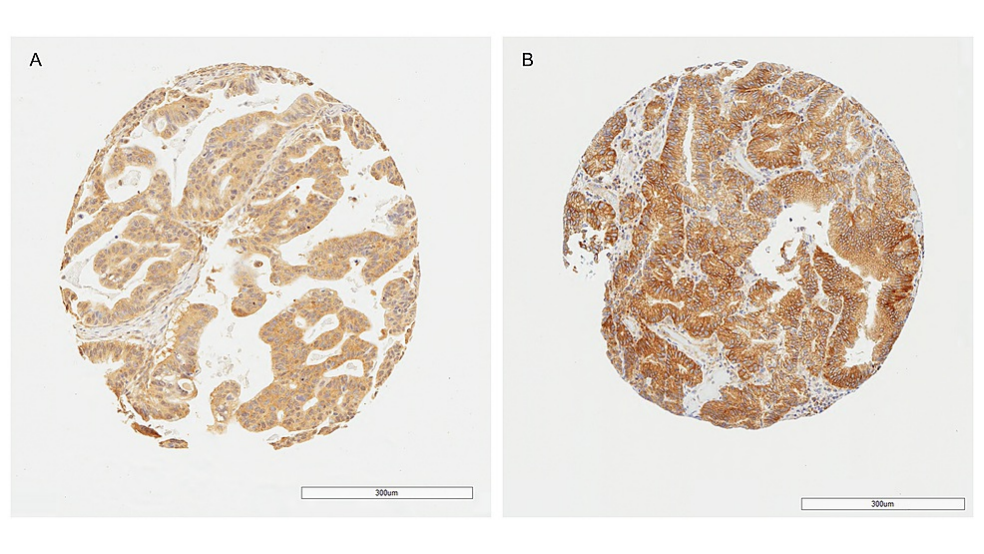
Immunohistochemical localization of proteins in an EAC tumor cell (original magnification x200) a. ASCL2 (cytoplasmic); b. LGR5 (cytoplasmic and membranous) ASCL2, Achaete scute-like 2; LGR5, Leucine-rich-repeat-containing G-protein-coupled receptor 5; EAC, esophageal adenocarcinoma

Twenty cases were ASCL2^low^ and 44 cases were ASCL2^high^. The representative picture of ASCL2^high^ is shown in Figure 2a. High ASCL2 expression was associated with a more significant number of positive lymph nodes (p=0.027). No association was observed between ASCL2 expression and age, sex, pathological stage, neoadjuvant therapy, or histological differentiation.

**Figure 3:**
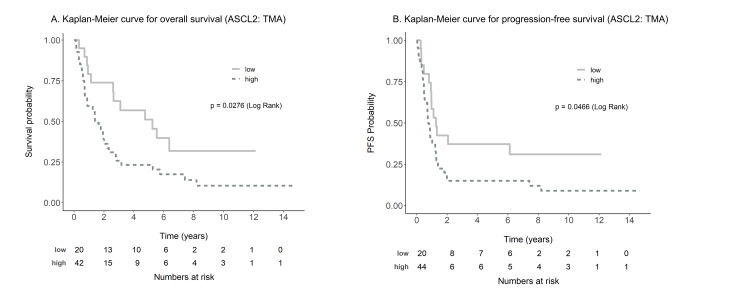
Survival analysis of 64 esophageal adenocarcinoma patients by Kaplan-Meier curve A. The overall survival rate in patients with high ASCL2 protein expression (dashed line) was significantly lower than that in patients with low ASCL2 expression (solid line), B. Progression-free survival in patients with high ASCL2 protein expression (dashed line) was significantly lower than that in patients with low ASCL2 expression (solid line) ASCL2, Achaete scute-like 2; TMA, tissue microarray; PFS, progression-free survival

For the cases in TMA, the median overall survival (OS) time was two years (95% CI, 1.4-3.2), and progression-free survival (PFS) time was 0.9 years (95% CI, 0.7-1.3). The median OS time for ASCL2^high^ was 1.4 years (95% CI, 0.9-2.3) compared to the much longer OS time for ASCL2^low^ at 5.2 years (95% CI, 2.6-8.2). The results of the KM analysis for OS are shown in Figure 3A (p=0.0276). Figure 3B shows the significant association of ASCL2 with PFS. The median PFS time for ASCL2^high^ was 0.8 years (95% CI, 0.5-1.3), compared to the 1.3 years for ASCL2^low^ (95% CI 1-8.2, p=0.0466). The results of the univariate and multivariate analyses for OS (Table 5) and PFS (Table 6) support the results of KM analysis. Of note, multivariate analysis indicated that ASCL2 was an independent predictor of prognosis in line with stage.

**Table 5:** Univariable and multivariable analysis of overall survival (tissue microarray) p<0.05 was considered statistically significant and is highlighted in *italics*. ASCL2, Achaete scute-like 2; LGR5, Leucine-rich-repeat-containing G-protein-coupled receptor 5; HR, hazard ratio; CI, confidence interval

	Covariate	HR (95% CI)	p-value	Global p-value
Univariable	n=62	46 events		
Age	Age	1.01 (0.99,1.03)		0.44
Gender	female	Reference		0.076
	male	2.32 (0.92,5.9)		
Histological grade	G1	Reference		0.3
	G2	0.97 (0.29,3.24)	0.97	
	G3	1.58 (0.46,5.44)	0.47	
Stage	I/II	Reference		0.001
	III	2.43 (1.04,5.7)	0.041	
	IV	5.62 (2.2,14.36)	<0.001	
Node positive		1.07 (1.03,1.11)		0.001
ASCL2 score	Low	Reference		0.031
	High	2.07 (1.07,4.02)		
LGR5 score	Low	Reference		0.4
	High	0.7 (0.31,1.58)		
Multivariable	n=62	46 events		
ASCL2 score	Low	Reference		0.011
	High	2.47 (1.23,4.95)		
Gender	Female	Reference		0.047
	Male	2.61 (1.01,6.72)		
Stage	I/II	Reference		0.001
	III	2.96 (1.23,7.13)	0.016	
	IV	8.15 (2.99,22.2)	0.001	

**Table 6:** Univariable and multivariable analysis of progression-free survival (tissue microarray) p<0.05 was considered statistically significant and the results were highlighted in *italics*. ASCL2, Achaete scute-like 2; LGR5, Leucine-rich-repeat-containing G-protein-coupled receptor 5; HR, hazard ratio; CI, confidence interval

	Covariate	HR (95% CI)	p-value	Global p-value
Univariable	n=64	50 events		
Age		1.00 (0.97,1.02)		0.84
Gender	Female	Reference		0.041
	Male	2.64 (1.04,6.7)		
Histological grade	G1	Reference		0.61
	G2	0.88 (0.26,2.89)	0.83	
	G3	1.18 (0.35,4.03)	0.79	
Stage	I/II	Reference		0.025
	III	2.05 (0.92,4.54)	0.078	
	IV	4.62 (1.89,11.32)	<0.001	
Node positive		1.06 (1.03,1.09)		<0.001
ASCL2 score	Low	Reference		0.05
	High	1.89 (1,3.57)		
LGR5 score	Low	Reference		0.62
	High	0.82 (0.37,1.82)		
Multivariable	n=64	50 events		
ASCL2 score	Low	Reference		0.038
	High	2.05 (1.04,4.02)		
Gender	Female	Reference		0.052
	Male	2.54 (0.99,6.5)		
Stage	I/II	Reference		<0.001
	III	2.23 (0.99,5.04)	0.054	
	IV	5.97 (2.31,15.41)	<0.001	

Fifty-six cases (88%) were LGR5^high^; eight cases (13%) were LGR5^low^. A representative picture of LGR5^high^ is shown in Figure 2b. Six out of 8 LGR5^low^ cases (75%) were pathological stage IV. LGR5^low^ had significantly more positive lymph nodes compared to LGR5^high^ (p=0.039). No other association was observed between LGR5 expression and other clinicopathological factors. In KM analysis, LGR5 was not a predictive factor of OS (LGR5^low^ 1.8 years 95% CI 0.7-2.8; LGR5^high^ 2 years 95% CI 0.7-7.4 p=0.393) nor PFS (LGR5^low^ 0.9 years 95% CI 0.5-1.4; LGR5^high^ 1.2 years 95%CI 0.5-14.7 p=0.62). Univariable Cox PH analyses also failed to show LGR5 significance in OS and PFS (Table 5-6: OS HR=1.27, 95% CI=0.52-3.09, p=0.59; PFS HR=1.69, 95% CI=0.67-4.21, p=0.26). No significant correlation was seen between the expressions of ASCL2 and LGR5 (p=0.42). Thirty-seven out of 64 cases (57.8%) were ASCL2^high^/LGR5^high^; 19 cases (29.7%) ASCL2^low^/LGR5^high^; seven cases (10.9%) ASCL2^high^/LGR5^low^; and one case (1.6%) ASCL2^low^/LGR5^low^.

ASCL2 expression is significantly increased in esophageal adenocarcinoma

**Figure 4:**
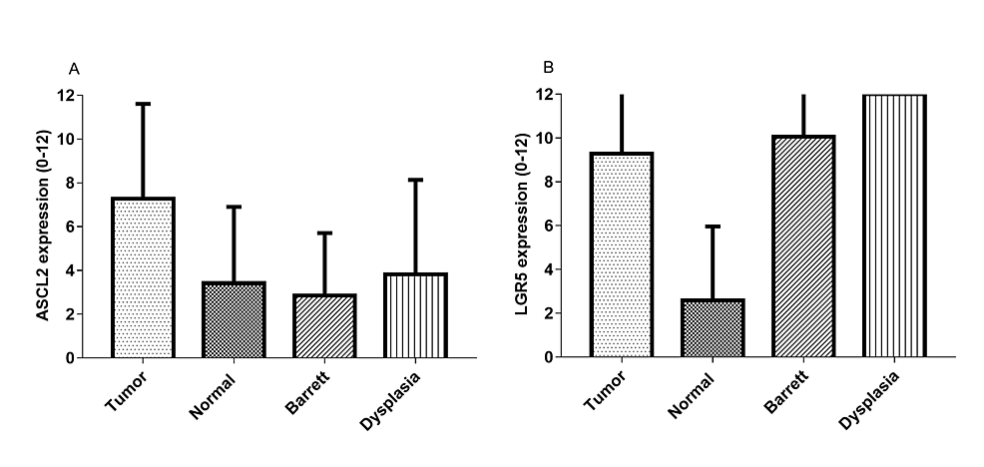
Immunohistochemical differential expression of ASCL2 and LGR5 in esophageal adenocarcinoma, normal (squamous epithelium), Barrett's esophagus (metaplastic glandular epithelium), and dysplasia (dysplastic glandular epithelium) A. ASCL2; B. LGR5 ASCL2, Achaete scute-like 2; LGR5, Leucine-rich-repeat-containing G-protein-coupled receptor 5; Normal, normal squamous epithelium; Barrett, Barrett's esophagus; Dysplasia, dysplastic glandular epithelium

Adjacent 43 normal squamous epithelium (normal), 22 metaplastic glandular epithelium (Barrett), and six dysplastic glandular epithelium (dysplasia) of the TMA patients were included in the TMA. EAC showed significantly increased ASCL2 expression compared to the normal (p<0.001) and Barrett (p<0.001) and a trend towards higher expression compared to dysplasia (p=0.0686, Figure 4A). Expression of LGR5 in the normal was significantly lower compared to the tumor (p<0.0001), Barrett (p<0.0001), and dysplasia (p<0.0001) but tumor, Barrett, and dysplasia showed similar expression (Figure 4B).

Further validation of ASCL2 in TCGA datasets

**Figure 5:**
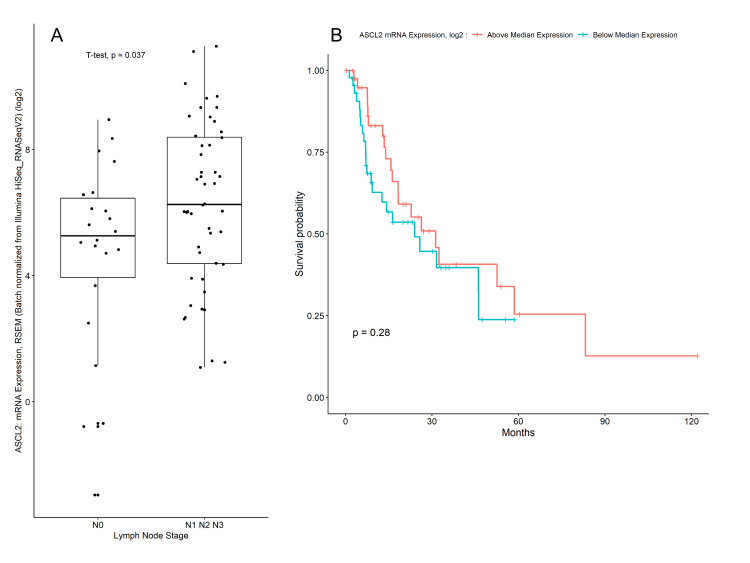
ASCL2 mRNA expression using the TCGA data set (A) The unpaired t-test shows patients with nodal status N0 have lower ASCL2 mRNA expression compared to patients with nodal status N1, N2, and N3, (B) Survival analysis of 86 esophageal adenocarcinoma patients by Kaplan-Meier curve ASCL2, Achaete scute-like 2; LGR5, Leucine-rich-repeat-containing G-protein-coupled receptor 5; TCGA, The Cancer Genome Atlas; RSEM, RNA-Seq by Expectation Maximization

The significance of ASCL2 protein expression in both WS and TMA prompted us to analyze the TCGA dataset. Eighty-six cases of EAC revealed that ASCL2 mRNA was correlated with nodal status. ASCL2 mRNA was not significantly correlated to survival using KM analysis (Figure 5). No mutation in the ASCL2 gene was identified in the TCGA dataset, but two cases showed deep deletion of the ASCL2 gene.

## Discussion

Here, we report the possible role of the ISC marker ASCL2 in EAC prognosis and carcinogenesis. The ability to appropriately classify cancer to specific subtypes is an effective strategy that EAC currently lacks, and immunohistochemistry remains the most available measure to detect biomarkers to this day [24]. In this study, we used immunohistochemistry to locate two key stem cell markers, ASCL2 and LGR5, and our results provided promising evidence that ASCL2 could potentially be used as a prognostic marker in EAC.

We examined the expression of ASCL2 using IHC in WS and TMA, as well as mRNA expression in TCGA datasets. The first important finding is that ASCL2 expression was significantly increased in EAC when compared with normal squamous epithelium and BE but not with dysplastic glandular epithelium. This finding implied an essential role of ASCL2 in the early development of EAC. EAC carcinogenesis is a multistep process, starting from gastroesophageal reflux, progressing to BE [25], and alteration in the Wnt pathway is implicated in the carcinogenesis of EAC, similar to other intestinal cancers [12]. ASCL2 is not only its direct transcriptional target but also the potent ISC fate determinator, working as a master regulator of LGR5 positive ISCs [14].

Patients with high ASCL2 expression showed worse survival in both WS and TMA compared with patients with low ASCL2 expression. According to our literature search, this is the first study to reveal the prognostic significance of ASCL2 in EAC. Of note, a high ASCL2 score had a significant impact on overall and progression-free survival in line with a pathological stage while other pathological factors, such as histological grade and the number of positive nodes, did not show a significant impact. ASCL2, a key player in tumor progression and metastasis, has been studied in other cancers, including colorectal cancer [26-27], consistently associated with poor prognosis. ASCL2 mRNA and protein using PCR assay and Western Blot Analysis in EAC cell lines have been studied by Zhao et al., who showed amplification and overexpression of ASCL2 [28]. Our bioinformatics analysis using TCGA data showed that ASCL2 mRNA was correlated to nodal status but not to OS or PFS; thus, further research is necessary to understand the mechanism of ASCL2 overexpression in EAC, with a particular focus on the Wnt pathway.

ISC markers have been extensively studied in colorectal cancer, where ASCL2 is known to control the fate of ISCs and colon cancer progenitor cells by regulating "stemness" genes such as LGR5 and controls self-renewal via R-spondin1/Wnt activation [15]. Further, ISCs impact the plasticity of epithelial-mesenchymal transition in colorectal cancer via the expression of microRNA-200 [29]. Contrarily, the impact of ISCs on the carcinogenesis of EAC has been studied to a much lesser extent. Only one study examined ASCL2 mRNA and protein overexpression in EAC cell lines, in which cells with stem cell-like features overexpressed ASCL2 [28], but to our knowledge, this study is the first to assess ASCL2 IHC expression in EAC. Becker et al. found LGR5 to be heterogeneously expressed in 24 cases of EAC WS using IHC, in which they concluded a high LGR5 score was associated with worse survival [30]. Our study also revealed heterogeneous staining of LGR5 in EAC WS; however, our results differed where LGR5 was not a significant prognostic marker in neither TMA nor WS. This discordance may be due to the heterogeneous IHC expression of LGR5 in EAC and may illustrate the difficulty in using LGR5 expression as a prognostic marker. While the existence of LGR5 stem cell-like cells in BE and EAC is mostly confirmed, its significance on cancer progression and metastasis remains to be determined with future studies.

This study has several limitations that should be taken into consideration. First of all, the sample size was relatively small for generalizing the clinical significance of the expression of ASCL2 and LGR5 in surgically resected EAC. Second, selection bias must be considered due to the retrospective nature of this study where no endoscopically resected cases were included, thus the number of Stage I cases is very small. Therefore, validation of these results using a large prospective data set is required to understand the significance of ISCs and the Wnt pathway in EAC carcinogenesis before it can be implemented in the clinical setting.

## Conclusions

The authors investigated ASCL2 as a potential biomarker of EAC based on known dual functions; (a) by promoting the Wnt signaling pathway and (b) regulating the ISCs located near the base of BE. Using immunohistochemistry, we suggest the possibility that high ASCL2 expression may be an independent prognostic factor for surgically resected EAC. Furthermore, our studies suggest that ASCL2 is implicated in the early stage of EAC carcinogenesis, which may provide additional evidence to the role of intestinal stem cells in Barrett's esophagus formation. Given several limitations of this retrospective study, further research is warranted to validate ASCL2 as a promising biomarker for predicting EAC survival and a key to understanding the role of intestinal stem cells in EAC carcinogenesis.
